# *N*-alkynyl derivatives of 5-fluorouracil: susceptibility to palladium-mediated dealkylation and toxigenicity in cancer cell culture

**DOI:** 10.3389/fchem.2014.00056

**Published:** 2014-07-29

**Authors:** Jason T. Weiss, Craig Fraser, Belén Rubio-Ruiz, Samuel H. Myers, Richard Crispin, John C. Dawson, Valerie G. Brunton, E. Elizabeth Patton, Neil O. Carragher, Asier Unciti-Broceta

**Affiliations:** ^1^Edinburgh Cancer Research UK Centre, MRC Institute of Genetics and Molecular Medicine, University of EdinburghEdinburgh, UK; ^2^MRC Human Genetics Unit, MRC Institute of Genetics and Molecular Medicine, University of EdinburghEdinburgh, UK

**Keywords:** chemotherapeutics, palladium, prodrugs, bioorthogonal chemistry, 5-fluorouracil

## Abstract

Palladium-activated prodrug therapy is an experimental therapeutic approach that relies on the unique chemical properties and biocompatibility of heterogeneous palladium catalysis to enable the spatially-controlled *in vivo* conversion of a biochemically-stable prodrug into its active form. This strategy, which would allow inducing local activation of systemically administered drug precursors by mediation of an implantable activating device made of Pd^0^, has been proposed by our group as a way to reach therapeutic levels of the active drug in the affected tissue/organ while reducing its systemic toxicity. In the seminal study of such an approach, we reported that propargylation of the *N*1 position of 5-fluorouracil suppressed the drug's cytotoxic properties, showed high stability in cell culture and facilitated the bioorthogonal restoration of the drug's pharmacological activity in the presence of extracellular Pd^0^-functionalized resins. To provide additional insight on the properties of this system, we have investigated different *N*1-alkynyl derivatives of 5-fluorouracil and shown that the presence of substituents near the triple bond influence negatively on its sensitivity to palladium catalysis under biocompatible conditions. Comparative studies of the *N*1- vs. the *N*3-propargyl derivatives of 5-fluorouracil revealed that masking each or both positions equally led to inactive derivatives (>200-fold reduction of cytotoxicity relative to the unmodified drug), whereas the depropargylation process occurred faster at the *N*1 position than at the *N*3, thus resulting in greater toxigenic properties in cancer cell culture.

## Introduction

Based on the ability to interfere with and/or halt cell division at different stages, cytotoxic agents of various classes have been used as chemotherapeutic drugs in antineoplastic treatment regimens for over 70 years (Chabner and Roberts, 2005; DeVita and Chu, 2008). Although highly effective, the mode of action of these drugs also renders them particularly harmful to healthy tissues with a high rate of cell regeneration. As a result, the clinical dose of most cytotoxic drugs is limited by their lack of selectivity for cancer cells (Chabner and Roberts, 2005; DeVita and Chu, 2008). To reinvigorate the medical use of approved drugs without a satisfactory safety profile and promising drug candidates that failed in clinical trials due to pharmacokinetic issues, one of the main strategies followed by medicinal chemists is to transform chemotherapeutic agents into latent prodrugs that become active in specific organs or tissues by a biological / metabolic mediator (Huttunen et al., 2008; Rautio et al., 2008).

Many different classes of prodrugs have been developed to date (Huttunen et al., 2008; Rautio et al., 2008), resulting in clinically approved therapeutics (e.g., the dopamine precursor levodopa Jenner, 2008) and numerous prodrug candidates and advanced technologies currently in preclinical and clinical development (such as directed enzyme prodrug therapies, which are based on the metabolic activation of drug precursors through enzymes artificially introduced into the organism Xu and McLeod, 2001). While most popular prodrugs become active through a biochemical process, significant progress on the use of benign non-biological means to activate drug precursors (Castano et al., 2006; Versteegen et al., 2013; Clavel et al., 2014; Velema et al., 2014; Weiss et al., 2014a,b; Zanda, 2014) mandate the consideration of expanding the classical prodrug concept and the distinction between two fundamentally different classes of prodrugs: biolabile and bioorthogonal prodrugs. Since the term biolabile is used to define compounds prone to transformation by biological means, the first class would represent all drug precursors belonging to the classical definition of prodrug (Huttunen et al., 2008; Rautio et al., 2008). On the other hand, inspired by the concept of bioorthogonality developed by Bertozzi in the early 2000's (Saxon and Bertozzi, 2000; Agard et al., 2004; Sletten and Bertozzi, 2011), bioorthogonal prodrugs would encompass physiologically-stable drug precursors subject to activation by non-native, non-biological, non-perturbing means, such as benign light sources (Castano et al., 2006; Velema et al., 2014), metal-free click chemistry (Versteegen et al., 2013), mild hyperthermia (Clavel et al., 2014) or bioorthogonal organometallic (BOOM) reactions (Weiss et al., 2014a,b; Zanda, 2014).

As a first-in-class prodrug approach, our group is investigating the application of metallic palladium as an activating device to modulate the cytotoxic activity of antineoplastic drugs in a bioorthogonal, spatially-controlled manner. The strategy is based on the unique catalytic properties and biocompatibility of heterogeneous Pd^0^ both *in vitro* and *in vivo* (Yusop et al., 2011; Unciti-Broceta et al., 2012; Weiss et al., 2014a,b), which has enabled the *in situ* BOOM activation of precursors of 5-fluorouracil (5FU) and gemcitabine in cancer cell culture (Weiss et al., 2014a,b). The surgical insertion of benign palladium-functionalized implants in the disease area (e.g., advanced tumors) in combination with the general administration of palladium-labile prodrugs, could not only decrease systemic levels of the active drug (thereby reducing unwanted toxicity in healthy tissues and organs), but also enhance cancer treatment by generating greater drug levels localized at the disease site than could ever be safely reached by systemic chemotherapy.

A palladium-labile prodrug needs to be designed in such a manner that it only undergoes chemical activation by mediation of this metal. Consequently, the nature of the chemical group used to mask the drug together with the position to be functionalized, are essential features which will determine the overall success of the strategy. In practice, based on the drug's mode of action and the selective catalytic properties of Pd^0^, masking strategies are designed to accomplish three goals: (i) eliminating drug's pharmacological properties; (ii) minimizing prodrugs' susceptibility to enzymatic cleavage; and (iii) rendering them “cleavable” by palladium catalysis.

**Figure 1:**
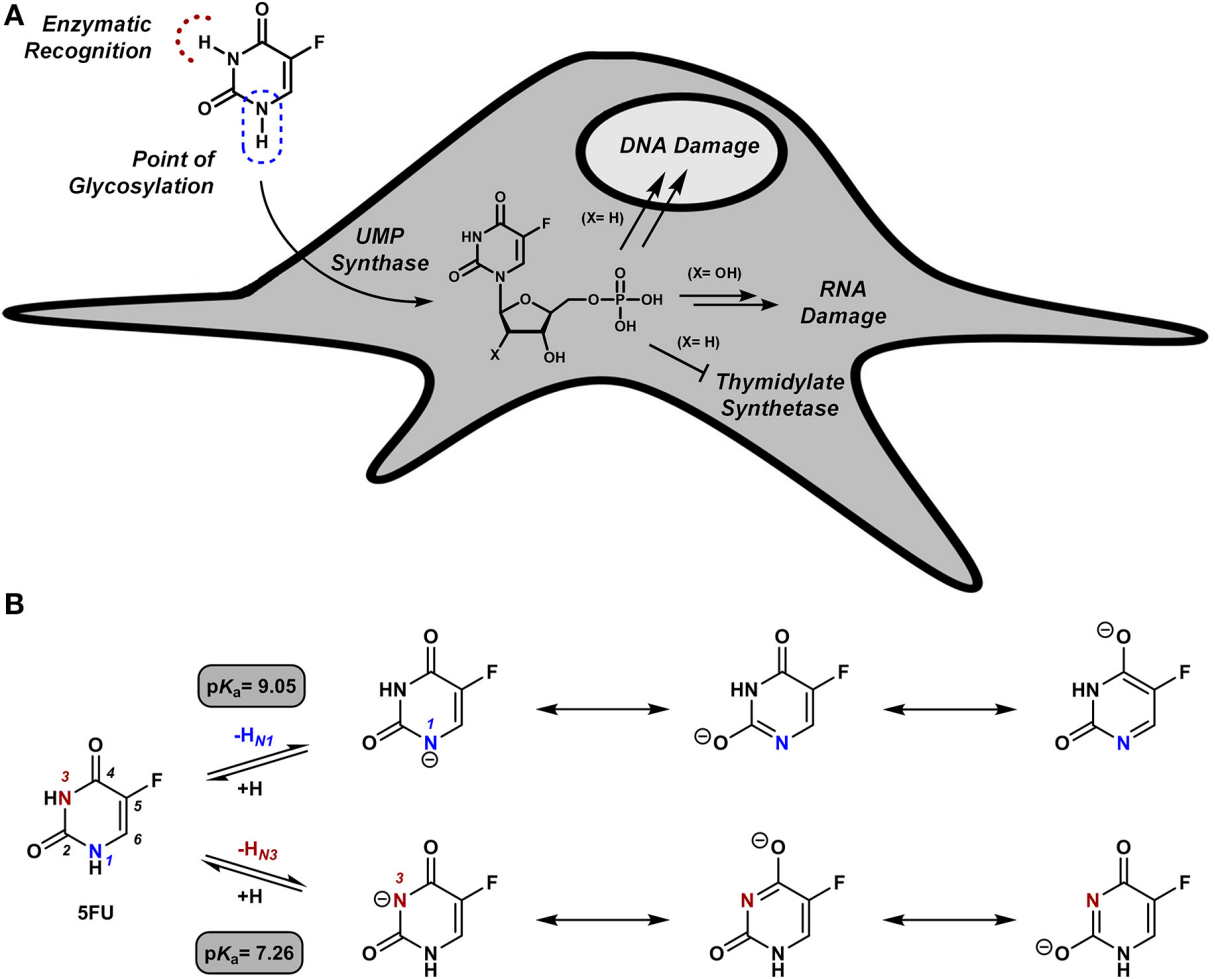
**(A)** Intracellular bio-functionalization of 5FU to generate cytotoxic metabolites. **(B)** 5FU its conjugate bases and their theoretical p*K*_a_ values (Jang et al., 2001).

Due to its long clinical history, its well-established mode of action and its narrow therapeutic index (Longley et al., 2003), cytotoxic 5FU was our first choice to explore this challenging strategy (Weiss et al., 2014a). 5FU is as an antimetabolite that undergoes intracellular metabolization on the NH group in position 1 to generate cytotoxic nucleotides (Figure 1A), which are the direct cause of 5FU pharmacological activity (Longley et al., 2003). Blocking the formation of active 5FU metabolites by functionalization of the *N*1 position was thus the rationale followed to chemically mask the cytotoxic properties of the drug. Moreover, due to 5FU's lactam/lactim tautomery (see Figure 1B), the *N*1 position of the ring possesses a p*K*_a_ value of ~9 (Jang et al., 2001), an unusually low value for NH groups that prompted us to explore chemical groups uncharacteristic for NH protection chemistries. The use of allyl, propargyl and benzyl moieties [alkyl groups widely employed in protection strategies for phenolic OH (p*K*_a_ ~ 9) (Weiss et al., 2014a)] was investigated on the basis on their anticipated biochemical stability and their potential lability in the presence of palladium (Weiss et al., 2014a). As expected, inactive prodrugs were successfully obtained by alkylation with each of the three groups employed. Remarkably, only the propargyl derivative displayed high sensitivity to palladium in biocompatible conditions, allowing the chemical rescue of 5FU's pharmacological activity in cancer cell culture by the mediation of extracellular Pd^0^-functionalized resins.

Herein we report a follow-on study to provide additional insights on the properties, scope and optimization of this novel prodrug system. A set of *N*1-alkynyl derivatives of 5FU have been developed and their sensitivity to palladium catalysis tested under biocompatible conditions. In addition, biological studies have been carried out to determine the difference in bioorthogonality and palladium sensitivity of the *N*1- vs. the *N*3-propargyl derivatives of 5FU.

## Materials and methods

### General methods

Chemicals and solvents were purchased from Fisher Scientific, Sigma-Aldrich or VWR International Ltd. NMR spectra were recorded at ambient temperature on a 500 MHz Bruker Avance III spectrometer. Chemical shifts are reported in parts per million (ppm) relative to the solvent peak. *Rf* values were determined on Merck TLC Silica gel 60 F254 plates under a 254 nm UV source. Purifications were carried out by flash column chromatography using commercially available silica gel (220–440 mesh, Sigma-Aldrich).

### Synthesis of Pd^0^-resins

Pd^0^-functionalized resins were prepared from NovaSyn TG amino resin HL (0.39 mmol NH_2_/g) as previously described (Weiss et al., 2014a).

### Synthesis of 5-fluoro-1-propargyluracil (3a)

Compound **3a** was prepared from 5-fluorouracil, **1**, as previously described (Weiss et al., 2014a).

### Synthesis of *N*1-functionalized 5FU derivatives 3b-e

5FU (100 mg, 0.77 mmol) and DBU (115 μl, 0.77 mmol) were dissolved in acetonitrile (2 ml), and the mixture was cooled down to 4°C in an ice bath. The corresponding alkyl bromide (0.77 mmol) was added dropwise and the reaction mixture allowed to warm up to room temperature. The mixture was stirred overnight, the solvents removed *in vacuo* and the resulting crude purified via flash chromatography (eluent: 1.5% MeOH in DCM), to yield compounds **3b-e** as pure white solids.

### 1-(1-butyn-3-yl)-5-fluorouracil (3b)

75 mg, 54% yield. *R_f_* = 0.55 (10% MeOH in DCM). ^1^H NMR (500 MHz, DMSO) δ 11.89 (s, 1H), 8.15 (*d, J* = 6.8, 1H), 5.40–5.30 (*m*, 1H), 3.61 (*d, J* = 2.4, 1H), 1.47 (*d, J* = 7.0, 3H). ^13^C NMR (126 MHz, DMSO) δ 157.01 (*d, J* = 26.0, C), 148.69, 140.19 (*d, J* = 231.4, C), 125.97 (*d, J* = 33.8, CH), 81.34, 76.53 (CH), 42.92 (CH), 20.47 (CH_3_). MS (ESI) m/z 181.0 [M-H]^−^. HRMS (FAB) m/z calcd for C_8_H_6_O_2_N_2_F_1_, 181.0412; found 181.0419.

### 1-(2-butyn-1-yl)-5-fluorouracil (3c)

63 mg, 45% yield. *R_f_* = 0.53 (10% MeOH in DCM). ^1^H NMR (500 MHz, DMSO) δ 11.86 (*s*, 1H), 8.11 (*d, J* = 6.7, 1H), 4.41 (*q, J* = 2.3, 2H), 1.82 (*t, J* = 2.4, 3H). ^13^C NMR (126 MHz, DMSO) δ 157.30 (*d, J* = 25.9, C), 149.04, 139.71 (*d, J* = 230.2, C), 128.93 (*d, J* = 33.8, CH), 81.61, 73.39, 37.30 (CH_2_), 3.07 (CH_3_). MS (ESI) m/z 181.0 [M-H]^−^. HRMS (FAB) m/z calcd for C_8_H_6_O_2_N_2_F_1_, 181.0412; found 181.0419.

### 1-(2-pentyn-1-yl)-5-fluorouracil (3d)

56 mg, 37% yield. *R_f_* = 0.53 (10% MeOH in DCM). ^1^H NMR (500 MHz, DMSO) δ 11.86 (s, 1H), 8.10 (*d, J* = 6.6, 1H), 4.43 (*t, J* = 2.2, 2H), 2.21 (*qt, J* = 7.5, 2.2, 2H), 1.06 (*t, J* = 7.5, 3H). ^13^C NMR (126 MHz, DMSO) δ 157.31 (*d, J* = 25.9, C), 149.03, 139.71 (*d, J* = 230.2, C), 128.87 (*d, J* = 33.7, CH), 87.05, 73.53, 37.27 (CH_2_), 13.45 (CH_3_), 11.60 (CH_2_). MS (ESI) m/z 195.0 [M-H]^−^. HRMS (FAB) m/z calcd for C_9_H_8_O_2_N_2_F_1_, 195.0573; found 195.0575.

### 1-(3-phenyl-1-propargyl)-5-fluorouracil (3e)

66 mg, 35% yield. *R_f_* = 0.66 (10% MeOH in DCM). ^1^H NMR (500 MHz, DMSO) δ 11.92 (*s*, 1H), 8.22 (*d, J* = 6.6, 1H), 7.49–7.36 (*m*, 5H), 4.73 (*s*, 2H). ^13^C NMR (126 MHz, DMSO) δ 157.35 (*d, J* = 25.9, C), 149.12, 139.83 (*d, J* = 230.4, C), 131.53 (CH), 129.01 (*d, J* = 34.0, CH), 129.04 (CH), 128.69 (CH), 121.60, 84.38, 83.79, 37.64 (CH_2_). MS (ESI) m/z 243.0 [M-H]^−^. HRMS (FAB) m/z calcd for C_13_H_8_O_2_N_2_F_1_, 243.0575; found 243.0574.

### Synthesis of 5-fluoro-3-propargyluracil (6)

#### N-^t^Boc protection of N1 position

5FU (100 mg, 0.77 mmol) was dissolved in a 2:1 mixture of acetonitrile and DMF (3 ml). Boc_2_O (252 mg, 1.16 mmol) and DMAP (19 mg, 0.15 mmol) were subsequently added to the mixture and stirred overnight at room temperature. The solvents removed *in vacuo* and the resulting crude purified via flash chromatography (eluent: hexane/ EtOAc 3:1), to yield compound **4** as a white solid (70 mg, 40%). ^1^H NMR (500 MHz, DMSO) δ 10.60 (*s*, 1H), 7.64 (*d, J* = 4.7, 1H), 1.62 (*s*, 9H). ^13^C NMR (126 MHz, DMSO) δ 159.21 (*d, J* = 24.4, C), 150.70, 139.59 (*d, J* = 224.8, C), 123.02 (*d, J* = 31.6, CH), 61.45, 29.35 (CH_3_).

#### Propargylation of N3 position

*N*1-^t^Boc-protected compound **4** (56 mg, 0.24 mmol), propargyl bromide (31 μl, 0.29 mmol) and DBU (55 μl, 0.36 mmol) were dissolved in dry DCM (2 ml), and the mixture stirred at room temperature for 4 h. The solvents were removed *in vacuo* and the reaction crude purified via flash chromatography (eluent: hexane / EtOAc 5:1), to yield compound **5** as an colorless oil (43 mg, 67%). ^1^H NMR (500 MHz, CDCl_3_) δ 7.40 (*d, J* = 4.6, 1H), 4.45 (*d, J* = 2, 2H), 2.49 (*t, J* = 2.6, 1H), 1.68 (*s*, 9H). ^13^C NMR (126 MHz, CDCl_3_) δ 159.08 (*d, J* = 24.3, C), 150.31, 140.51 (*d, J* = 233.9, C), 123.13 (*d, J* = 33.9, CH), 76.22, 75.88 (CH), 64.18, 38.01 (CH_2_), 29.76 (CH_3_).

#### N1-Boc deprotection

Compound **5** (28 mg, 0.1 mmol) and K_2_CO_3_ (7 mg, 0.05 mmol) were dissolved in MeOH (2 ml), and the mixture stirred at room temperature for 3 h. The solvents were removed *in vacuo* and the resulting crude purified via flash chromatography (eluent: 3% MeOH in DCM), to yield compounds **6** as a colorless solid (12 mg, 71%). ^1^H NMR (500 MHz, MeOD) δ 7.61 (*d, J* = 5.2, 1H), 4.64 (*dd, J* = 2.5, 0.5, 2H), 2.57 (*t, J* = 2.5, 1H). ^13^C NMR (126 MHz, MeOD) δ 158.82 (*d, J* = 25.8, C), 151.06, 141.35 (*d, J* = 229.9, C), 125.88 (*d, J* = 32.1, CH), 78.65, 71.94 (CH), 31.06 (CH_2_). MS (ESI) m/z 167.0 [M-H]^−^. HRMS (FAB) m/z calcd for C_7_H_4_O_2_N_2_F_1_, 167.0262; found 167.0252.

### Synthesis of 1,3-dipropargyl-5-fluorouracil (7)

5FU (100 mg, 0.8 mmol) and DBU (345 μl, 2.3 mmol) were dissolved in dry DMF (2 ml) under a nitrogen atmosphere, and the mixture was cooled down to 4°C in an ice bath. Propargyl bromide (170 μl, 1.6 mmol) was added dropwise and the reaction mixture allowed to warm up to room temperature. The mixture was stirred overnight, the solvents removed *in vacuo* and the resulting crude purified via flash chromatography (eluent: 1.5% MeOH in DCM), to yield compounds **7** as a colorless solid (136 mg, 86%). ^1^H NMR (500 MHz, CDCl_3_) δ 7.60 (*d, J* = 5.3, 1H), 4.72 (*d, J* = 2.4, 2H), 4.61 (*d, J* = 2.6, 2H), 2.56 (*t, J* = 2.6, 1H), 2.20 (*t, J* = 2.5, 1H). ^13^C NMR (126 MHz, CDCl_3_) δ 156.19 (*d, J* = 26.0, C), 148.92, 140.26 (*d, J* = 237.3, C), 125.33 (*d, J* = 33.7, CH), 77.24, 76.74 (CH), 75.47, 71.51 (CH), 38.18 (CH_2_), 31.27 (CH_2_). MS (ESI) m/z 435.2 [2M+Na]^+^. HRMS (FAB) m/z calcd for C_10_H_6_O_2_N_2_F_1_, 205.0419; found 205.0414.

### Pd^0^-mediated 5FU synthesis in biocompatible conditions

Prodrugs **3a-e, 6** and **7** (100 μM in DMSO) were dissolved in PBS (1 mL) with 1 mg of Pd^0^-resins and shaken at 1,200 rpm and 37°C in a Thermomixer. Reaction crudes were monitored at 0, 6, and 24 h by analytical HPLC (Agilent) using the UV detector at 280 nm to avoid the detection of PBS salts. Eluent A: water and formic acid (0.1%); eluent B: acetonitrile, formic acid (0.1%); *A*/*B* = 95: 5 to 5: 95 in 3 min, isocratic 1 min, 5: 95 to 95: 5 in 1 min, isocratic 1 min.

#### Study of the influence of pH in the conversion rate

The pH of the PBS buffer was adjusted with 1 N solutions of hydrochloric acid or sodium hydroxide using a pH meter (Mettler Toledo). Reactions and analyses were carried out at pH 6.5, 7.0, and 7.5 as described above.

### Cell culture

Cell lines were grown in culture media supplemented with serum (10% FBS) and L-glutamine (2 mM) and incubated in a tissue culture incubator at 37°C and 5% CO_2_. Human pancreas adenocarcinoma BxPC-3 cells (a kind gift from Dr Mark Duxbury) were cultured in Roswell Park Memorial Institute (RPMI) media. Human breast adenocarcinoma MCF7 cells (purchased from ATCC), human ovarian carcinoma PE04 cells (a kind gift from Prof Charlie Gourley), human colorectal carcinoma HCT116 cells (a kind gift from Dr Van Schaeybroeck) and human breast cancer R-SKBR3a cells were all cultured in Dulbecco's Modified Eagle Media (DMEM).

### Cell viability studies

HCT116 cells were seeded in a 96 well plate format at 1000 cells/well and incubated for 48 h before treatment. Each well was then replaced with fresh media containing compound **1, 3a, 6**, or **7** and incubated for 5 days. Untreated cells were incubated with DMSO (0.1% v/v). PrestoBlue™ cell viability reagent (10% v/v) was added to each well and the plate incubated for 1 h. Fluorescence emission was detected using a PerkinElmer EnVision 2101 multilabel reader (Perkin Elmer; excitation filter at 540 nm and emissions filter at 590 nm). All conditions were normalized to the untreated cells (100%) and curves fitted using GraphPad Prism using a sigmoidal variable slope curve.

### Time-lapse proliferation study of drug vs. boom activation assays of compounds 3a, 6, and 7

HCT116 cells were plated as described before and each well was then replaced with fresh media containing: Pd^0^-resins (0.67 mg/mL) with DMSO (0.1% v/v); **3a, 7**, or **6** (100 μM) with DMSO (0.1% v/v); 5FU **1** (100 μM) with DMSO (0.1% v/v); or combination of 0.67 mg/mL of Pd^0^-resins + **3a, 7**, or **6** (100 μM) with DMSO (0.1% v/v). Untreated cells were incubated with DMSO (0.1% v/v). Each well was imaged every 3 h over 5 d under standard incubation conditions using an IncuCyte™ ZOOM microscope (placed inside the incubator). Imaged-based analysis of cell confluence was carried out using the IncuCyte™ software.

### Pd^0^-mediated dealkylation of compounds 3a and 6 in cell culture

HCT116 cells, were plated as described above. BxPC-3 were plated at 2500 cell / well, MCF7 cells were plated at 2000 cells/well, PE04 cells were plated at 1000 cells / well and R-SKBR3a cells were plated at 156 cells/well. Each well was then replaced with fresh media containing: Pd^0^-resins (0.67 mg/mL) with DMSO (0.1% v/v); **3a**, or **6** (3, 10, 30, 100 μM) with DMSO (0.1% v/v); 5FU **1** (3, 10, 30, 100 μM) with DMSO (0.1% v/v); or combination of 0.67 mg/mL of Pd^0^-resins + **3a** or **6** (3, 10, 30, 100 μM) with DMSO (0.1% v/v). Untreated cells were incubated with DMSO (0.1% v/v). Cells were incubated with drugs for 5 days. PrestoBlue™ cell viability reagent (10% v/v) was added to each well and the plates were incubated between 60 and 180 min depending on the cell line. Fluorescence emission was detected and results normalized as described above.

## Results and discussion

### Pd^0^-functionalized resins as heterogeneous catalysts for boom chemistry

Based on the potential toxicity of palladium as a contaminant in the food chain, most of the early examples of palladium substrates employed in chemical biology were colorimetric and fluorogenic probes used to detect palladium in biological samples (Li et al., 2013). Nevertheless, our group (Yusop et al., 2011; Unciti-Broceta et al., 2012; Weiss et al., 2014a,b; Zanda, 2014) and others (Li et al., 2011, 2014; Michel et al., 2012; Spicer et al., 2012; Spicer and Davis, 2013) have recently shown that the catalytic properties of palladium are biocompatible and its cytotoxicity controllable to a certain degree, thus allowing the development of various BOOM reactions in cell culture. While soluble palladium species can display significant cytotoxic properties (Environmental Health Criteria, 2002), metallic palladium is the safest form of this transition metal (Environmental Health Criteria, 2002; Rushforth, 2004). On this basis, we investigated the development of solid devices functionalized with palladium nanoparticles as a way to eliminate the free mobility of palladium and induce spatially-controlled chemical reactions.

To mediate BOOM heterocatalysis outside cells, we developed a functional device consisting of palladium nanoparticles entrapped in a biocompatible polymer matrix. These palladium-functionalized resins (Pd^0^-resins) are larger than cells (150 μm in average diameter) and formed by a co-polymer matrix made of polyethylene glycol grafted onto polystyrene resin (Weiss et al., 2014a), two polymers that have been extensively employed in the manufacture of a variety of biomedical devices (Alcantar et al., 2000; Sanchez-Martin et al., 2005; Dhaliwal et al., 2011; Unciti-Broceta et al., 2011). As previously reported (Weiss et al., 2014a,b), we have demonstrated the high biocompatibility of these catalyst-entrapped polymeric structures both *in vitro* and *in vivo*. On this basis, Pd^0^-resins (containing 4.4% *w*/*w* in Pd) were used as the extracellular activating device in the studies subsequently described.

### Design, synthesis and Pd^0^-lability of *N*1-alkynyl derivatives of 5FU

**Figure 2:**
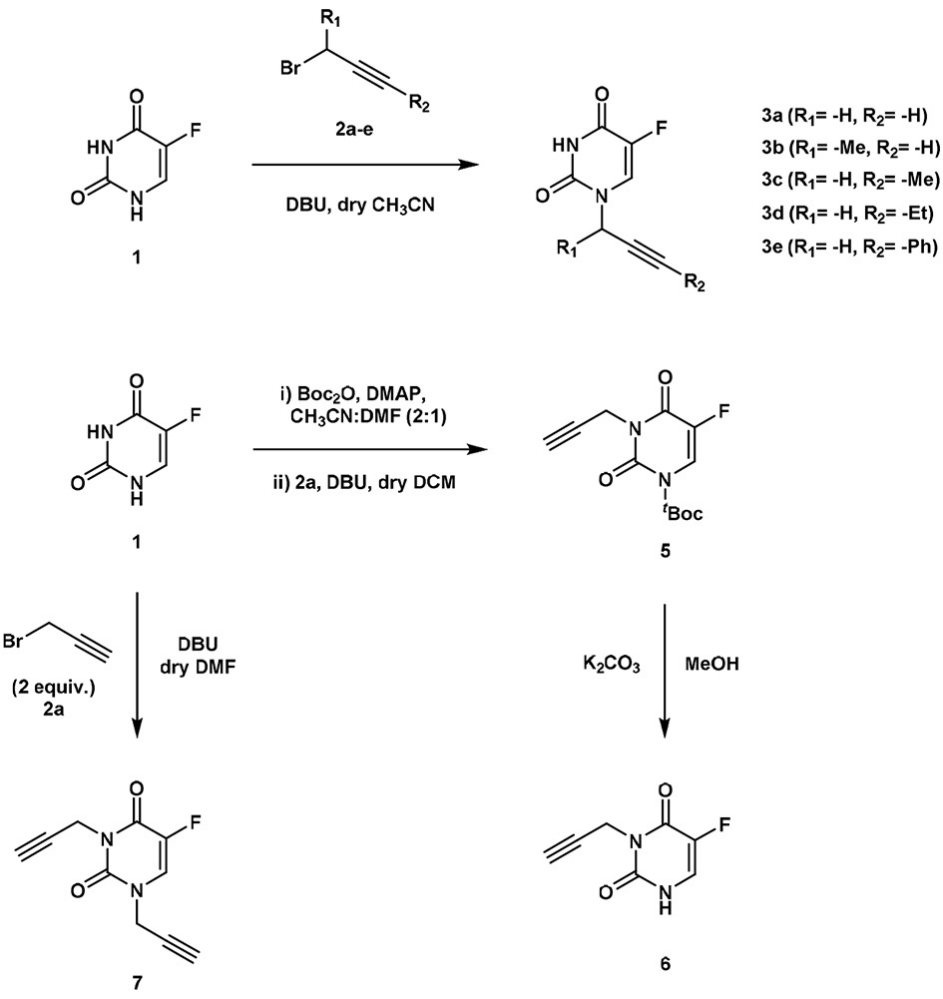
**Synthesis of compounds 3a-e (upper panel) and compounds 6, 7 (lower panel)**.

**Figure 3:**
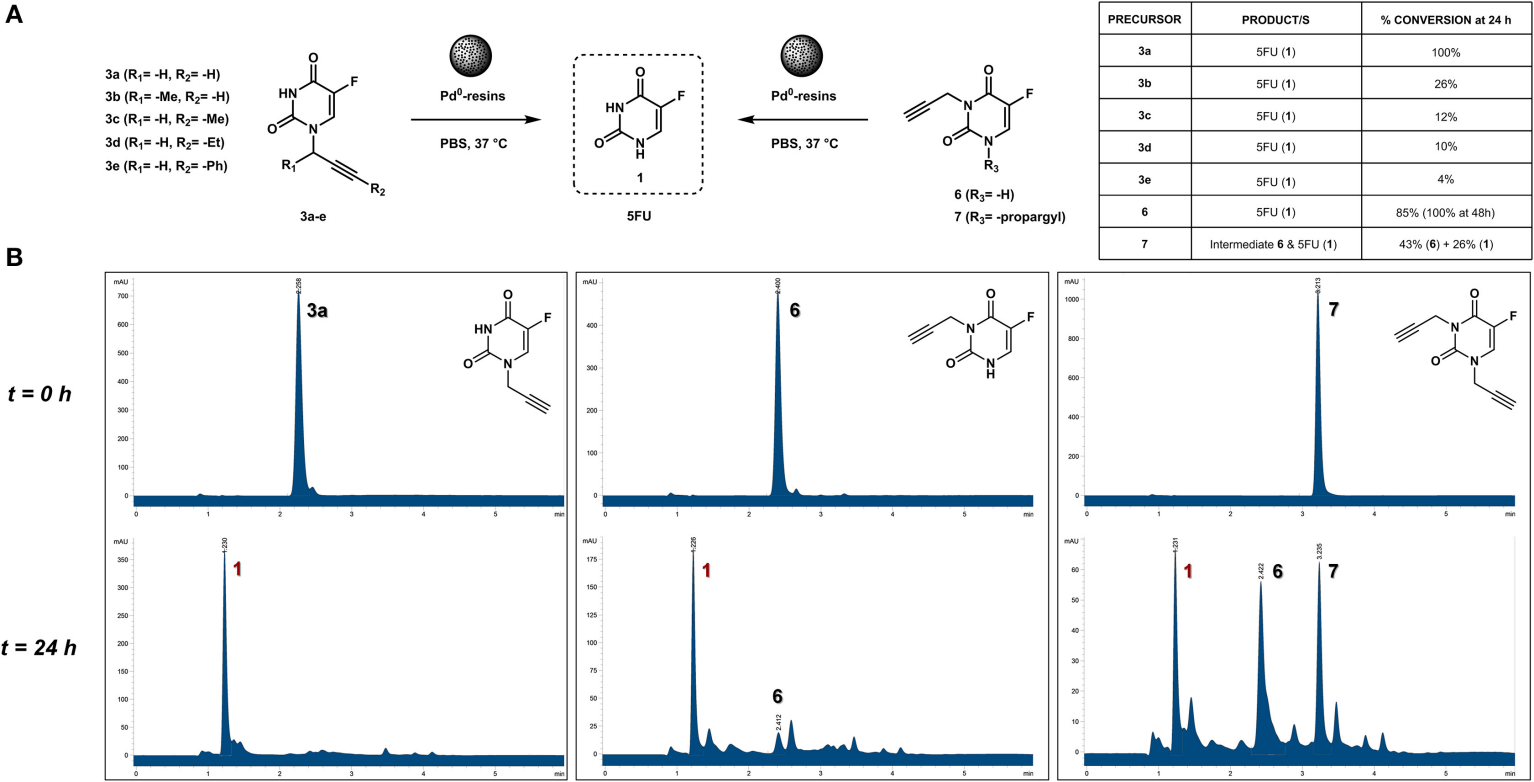
**(A)** Palladium-mediated dealkylation of compounds **3a-e, 6**, and **7** and table containing conversion percentages (relative values calculated by chromatographic peak integration). Each of the drug precursors (100 μM) were incubated with 1 mg/mL of Pd^0^-resins in PBS at 37°C for 24 h and the crude reaction analyzed by HPLC. **(B)** HPLC chromatograms (UV detector 280 nm) of 100 μM PBS solutions of compounds **3a** (left panel), **6** (central panel), and **7** (right panel) treated with Pd^0^-resins at 37°C for 0 h (top) and 24 h (bottom).

The clinical application and effective therapeutic response to several chemotherapeutics with a cytotoxic mechanism-of-action, including 5FU, is severely limited by numerous dose-limiting toxicities in patients. As previously reported (Weiss et al., 2014a), functionalization of the *N*1 position of 5FU with a propargyl group resulted in a bioorthogonal prodrug (**3a**) prone to conversion into 5FU in the presence of Pd^0^-resins both in PBS (biocompatible solution) and cell culture; a strategy that could allow for the reduction of systemic side effects of 5FU treatment. In order to investigate whether other alkynes could increase the rate of the dealkylation process, a set of *N*1-alkynyl derivatives of 5FU were synthesized following the procedure described in Figure 2. Subsequently, to study the susceptibility of derivatives **3b-e** to palladium catalysis, Pd^0^-resins were used as the heterogeneous catalyst and palladium-labile 5-fluoro-1-propargyluracil (**3a**) as positive control (Figure 3A). Compounds **3a-e** (100 μM) and Pd^0^-resins [1 mg/mL, (Pd^0^) = 400 μM] were dispersed in PBS (isotonic buffered solution at pH =7.4), incubated at 37°C for 24 h and the reaction crudes analyzed by HPLC using a UV detector. While compound **3a** led to 100% conversion in less than 24 h, compounds **3b-e** generated considerably lower levels of 5FU, **1** (see small table in Figure 3A). The susceptibility to palladium of compounds **3c-e** (containing a methyl, ethyl and benzyl group at the terminal carbon of the triple bond, respectively) was inversely proportional to the size of the moiety, indicating that the lesser the accessibility to the triple bond, the slower the reaction occurs. 24 h reaction of compound **3b** with Pd^0^-resins resulted in a 26% conversion into 5FU, a clear improvement over derivatives **3c-e** but significantly inferior to the reactivity of **3a** toward palladium. These results indicate that steric hindrance is a limiting factor in the reaction kinetics and, therefore, suggest that the non-substituted propargyl group is the optimal choice to generate palladium-labile bioorthogonal probes and prodrugs.

### *N*1- vs. *N*3-propargyl derivatives of 5FU: sensitivity to Pd^0^

Due to 5FU tautomerism, the NH groups at positions 1 and 3 possess relatively low p*K*_a_ values. Since, according to the literature (Jang et al., 2001), the *N*3 position has a lower p*K*_a_ value than the *N*1, we hypothesized that propargylation of the *N*3 position could in principle generate derivatives with improved sensitivity to palladium catalysis. Importantly, the absence of a free NH group in that position would impede the formation of hydrogen bonding interactions between the substrate and the active site of the enzyme (UMP synthase) (Wittmann et al., 2008), thus reducing the efficacy of the phosphorylation process and the generation of 5FU's cytotoxic nucleotides (Figure 1A). On this basis, bioorthogonal prodrugs could also be generated by chemical masking of that particular position. To investigate this, 5-fluoro-3-propargyluracil (**6**) and 1,3-dipropargyl-5-fluorouracil (**7**) were synthesized from 5FU (see Figure 2) and their susceptibility to palladium catalysis tested as described above (Figures 3A,B). Interestingly, palladium-mediated dealkylation of compound **6** underwent depropargylation at a slower rate than compound **3a**, with a conversion rate of approx. 85% after 24 h incubation. As observed in Figure 3B (right panel), 24 h reaction of bis-protected compound **7** with Pd^0^-resins resulted in the generation of 5FU (26%) and derivative **6** (43%), thus confirming that dealkylation of the propargyl group proceeds faster at the *N*1 position than the *N*3 one. While the theoretical p*K*_a_ values of each group (Jang et al., 2001) would have predicted the opposite outcome, the *N*3 is flanked by two oxo groups and the *N*1 by only one, underlining again the relevance of steric and conformational effects on the depropargylation kinetics.

### *N*1- vs. *N*3-propargyl derivatives of 5FU: influence of pH on the Pd^0^-mediated dealkylation of compounds 3a and 6

Due to hypoxia and glucose deprivation, solid tumors are estimated to have a pH in the order of 0.5 units lower than healthy tissues (Tannock and Rotin, 1989; Xu et al., 2002). Since the prodrug strategy proposed herein is expected to have application against locally-advanced tumors, it was suggested to examine the effect of pH changes in the reaction conversion rate. Hence, palladium-mediated depropargylation of compounds **3a** and **6** was carried out as above described at various pH (6.5, 7.0, and 7.5), and reactions analyzed by HPLC at different timepoints (6 and 24 h). As shown in Table 1, the pH had a noticeable effect on the conversion rates, particularly after short incubation periods. While propargyl cleavage of compound **3a** was completed in less than 24 h at each of the pH's tested, the conversion rate after 6 h was higher at a slightly basic pH (7.5). The same trend was observed for compound **6**. Importantly, even though the reaction is enhanced at slightly basic pH, this study demonstrates that the *N*-depropargylation process is compatible with the range of pH expected to be found *in vivo*.

**Table 1:** **Influence of pH in the palladium-mediated dealkylation of compounds 3a and 6**.

**Prodrug**	***t* = 6 h**			***t* = 24 h**		
	**pH = 6.5**	**pH = 7.0**	**pH = 7.5**	**pH = 6.5**	**pH = 7.0**	**pH = 7.5**
**3a**	28.9%	33.7%	44.4%	100%	100%	100%
**6**	21.3%	33.4%	39.1%	75.6%	76.9%	80.6%

Conversion percentages were calculated by chromatographic peak integration.

### *N*1- vs. *N*3-propargyl derivatives of 5FU: study of bioorthogonality

**Figure 4:**
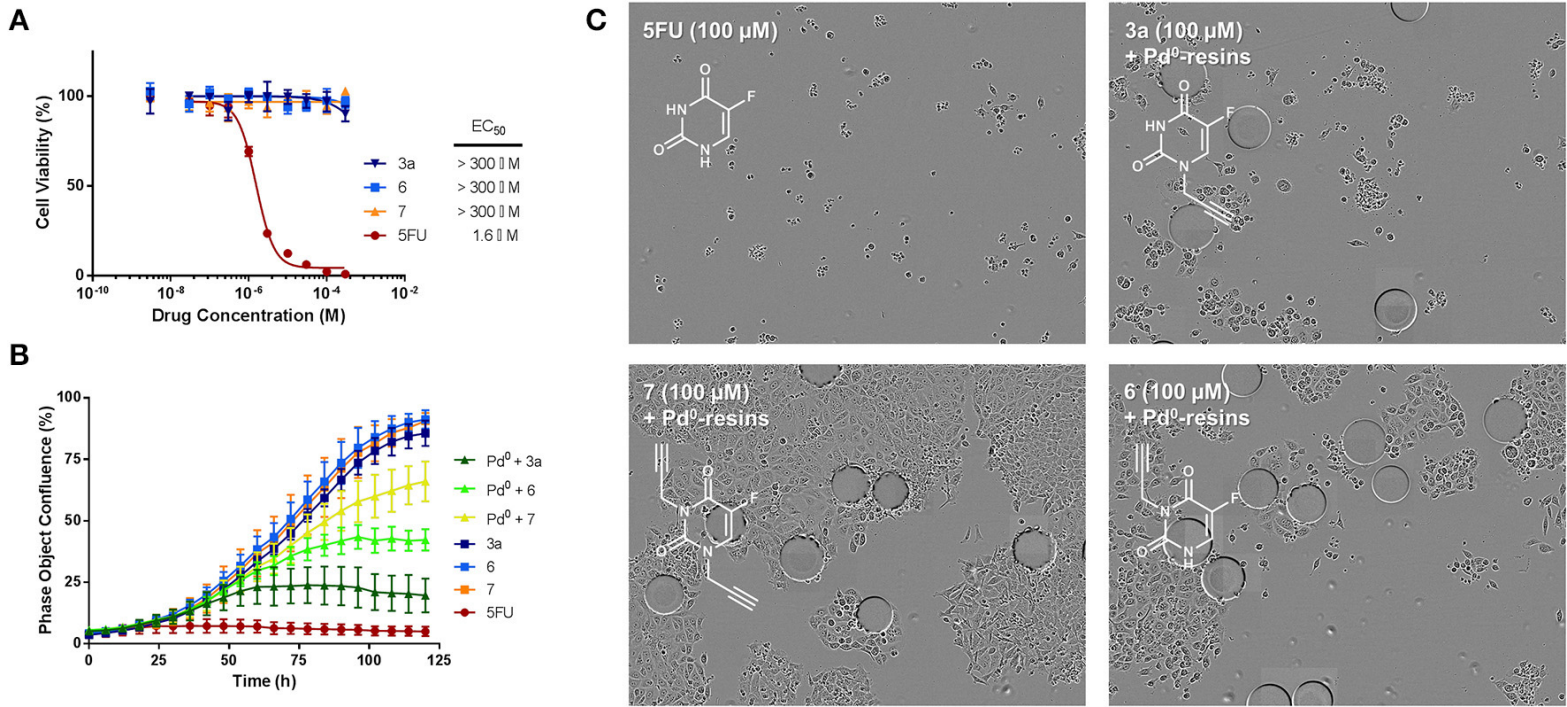
**(A)** Study of prodrugs' bioorthogonality. Semi Log dose response curves and calculated EC_50_ values of prodrugs **3a, 6**, and **7** in comparison to unmodified 5FU (**1**) in HCT116 cells. Cell viability was measured at day 5 using PrestoBlue™ reagent. Error bars: ± *SD* from *n* = 3. **(B)** Bioorthogonally-activated toxigenic effect in HCT116 cancer cell culture: Real-time cell confluence study. The cell population was monitored for 120 h using an IncuCyte ZOOM system in an incubator (5% CO2 and 37°C). Drug/prodrug concentration: 100 μM. Pd^0^-resins concentration: 0.67 mg/mL. Error bars: ± *SD* from *n* = 3. **(C)** Phase-contrast images of cells after 5 days of treatment with: 100 μM of 5FU (top left); 0.67 mg/mL Pd^0^-resins + 100 μM of **7** (bottom left); 0.67 mg/mL Pd^0^-resins + 100 μM of **3a** (top right); 0.67 mg/mL Pd^0^-resins + 100 μM of **6** (bottom right). Pd^0^-resins are identified as spheres of 150 μm (average diameter).

To evaluate whether the cytotoxic activity of the 5FU prodrugs were successfully masked, dose response viability studies were carried out with HCT116 colorectal cancer cells. Data analysis confirmed that, likewise for prodrug **3a**, compounds **6** and **7** did not display antiproliferative properties at any of the concentrations used (Figure 4A), thus confirming that propargylation of any NH group of 5FU result in the elimination of the drug's pharmacological properties.

### *N*1- vs. *N*3-propargyl derivatives of 5FU: Pd^0^-mediated prodrug activation in cancer cell culture

*In situ* generation of cytotoxic 5FU (**1**) from prodrugs **3a, 6** and **7** was first investigated with HCT116 cells in standard cell culture conditions using Pd^0^-resins as the extracellular activating device. Prodrugs (100 μM) and Pd^0^-resins (0.67 mg/mL) were incubated independently (negative controls) or in combination (BOOM activation assay), and unmodified 5FU (**1**) used as the positive control. Because each prodrug/Pd^0^-resin combination was expected to become phenotypically active at a different rate (see above studies), automated kinetic analysis of cell proliferation rate over time was used as a screening strategy to determine and rank the efficiency of distinct prodrug/Pd^0^-resin combinations. To enable the temporal visualization and quantification of the experiment, cell growth was monitored for 5 days by time-lapse imaging using an IncuCyte ZOOM device (Weiss et al., 2014a,b). To obtain the maximal differentiation among the experiments, the cell seeding density was optimized to reach confluency at day 5. While neither the prodrugs nor the Pd^0^-resins exhibited cytotoxicity, combinations of prodrugs with Pd^0^-resins displayed significant toxigenic effect (Figure 4B), confirming that the three prodrugs were bioorthogonally converted–at least partly– into cytotoxic 5FU (**1**). The enhanced sensitivity of prodrug **3a** to BOOM heterocatalysis was evidenced by a significantly smaller bell-shaped curve than those caused by either prodrug **6** and **7** in the presence of the palladium source. As shown in Figure 4C (phase contrast images of cells after 5 days of treatment), the antiproliferative effect of prodrugs **6** and **7** incubated with Pd^0^-resins was significantly lower than that of 5FU (**1**), whereas prodrug **3a** generated similar cytotoxic effect to the unmodified drug. In the presence of Pd^0^-resins, only compounds **3a** and **6** induced less than 50% of cell viability at 100 μM.

**Figure 5:**
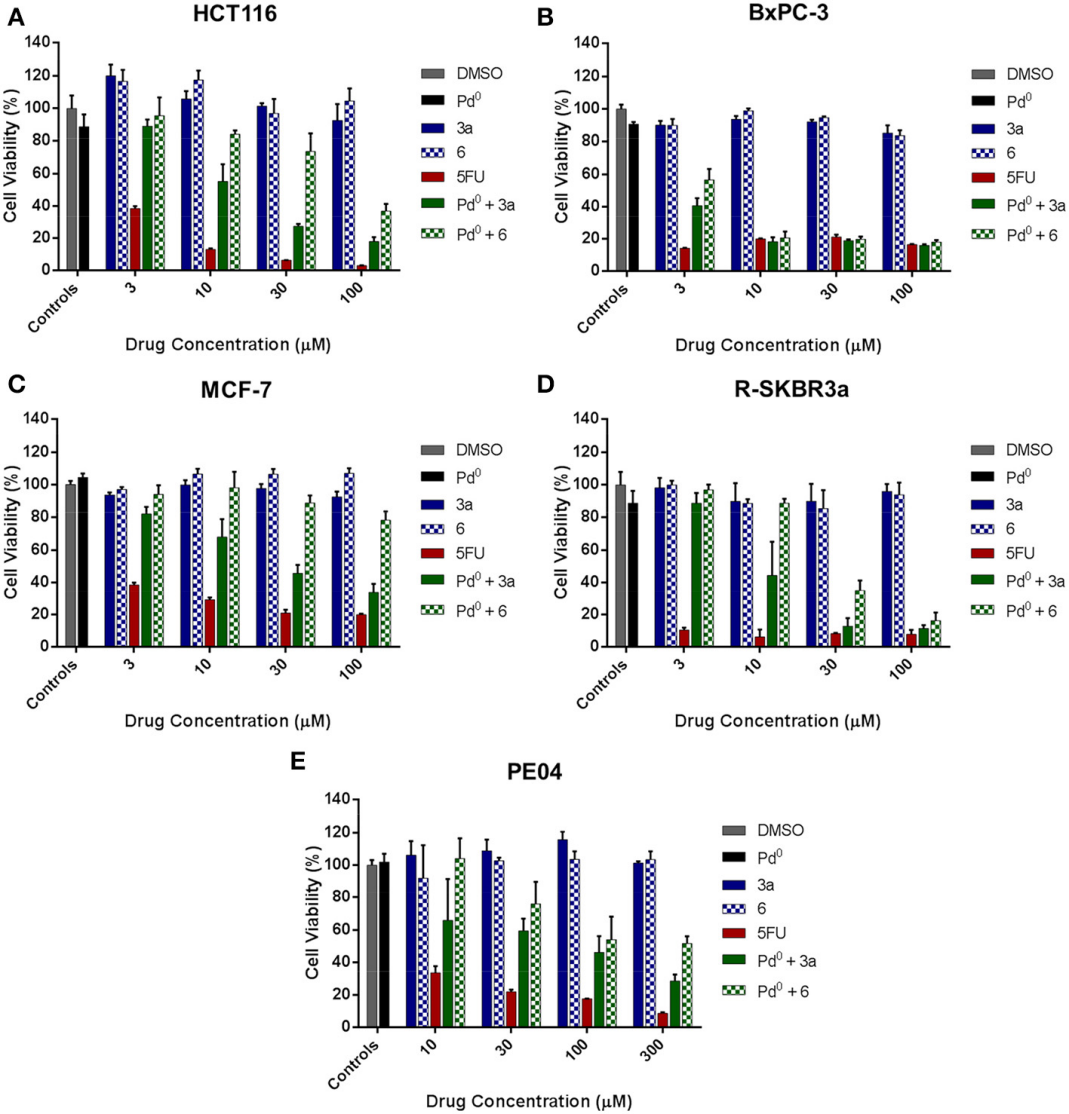
**Palladium-mediated conversion of prodrugs 3a and 6 into 5FU in cancer cell culture**. **(A)** Colorectal cancer HCT116 cells; **(B)** Pancretic adenocarcinoma BxPC-3 cells; **(C)** Breast cancer MCF-7; **(D)** Breast cancer R-SKBR3a cells; **(E)** Ovarian carcinoma PE04 cells. Drug generation was indirectly measured by analysis of cell viability after 5 days of treatment using the PrestoBlue™ Reagent (Life Technologies). Treatments: untreated cell control (0.1% *v*/*v* DMSO in media, negative control); Pd^0^-resins (0.67 mg/mL, negative control); 3–100 μM of **3a** or **6** (negative control); 3–100 μM of 5FU (positive control); and Pd^0^-resin (0.67 mg/mL) + **3a** or **6** (BOOM reaction assay).

On the basis of the toxigenicity demonstrated by prodrugs **3a** and **6** in combination with the heterogeneous palladium source, these prodrugs were selected for further exemplification of the strategy with a range of human cancer cell types, i.e., colorectal cancer HCT116 cells, pancreatic adenocarcinoma BxPC-3 cells, ovarian carcinoma PE04 cells and two types of breast cancer cell lines: ER-overexpressing MCF7 cells and R-SKBR3a cells, a derivative of HER2-overexpressing SKBR3 cells with induced resistance to AZD8931 (Creedon et al., in press). Once again, low cell seeding numbers were used to augment the effect of 5FU treatment and thus impose more discriminative conditions to the rate of drug generation. In accordance with previous observations, although both prodrugs **3a** and **6** showed high bioorthogonality in the absence of palladium at the range of concentrations used, prodrug **3a** proved again to possess enhanced susceptibility to palladium-mediated activation by displaying superior toxigenic effect in all the cell lines tested (Figure 5). Nevertheless, it is noteworthy that prodrug **3a**/and prodrug **6**/Pd^0^-resins combinations induced similar cytotoxic phenotype in pancreatic cancer BxPC-3 cells (Figure 5B). This is considered to be due to the high sensitivity of this cell line to 5FU treatment, thus requiring the generation of relatively low levels of drug to induce a strong antiproliferative effect. On the contrary, in the presence of cancerous cells with higher resistance to 5FU such as HCT116, MCF7, and PE04, the difference in the activation rate (= toxigenicity Weiss et al., 2014b) between both prodrug/activator combinations was highly discriminative, thus evidencing the lower toxigenic effect enabled by the prodrug **6**/Pd^0^-resins combination (Figures 5A,C,E). This study strongly indicates that the rate of activation is a key limiting factor for the translation of palladium-labile prodrugs into the clinic. To successfully achieve sustained and effective cytotoxic levels of drug and be able to control tumor growth, the kinetics of the activation process needs to superior to the rate of cancer cell proliferation.

## Conclusions

In conclusion, a set of 2-alkyn-1-yl groups (containing a triple bond in position 2 relative to the point of connection to 5FU) were used to synthesize, test and rank palladium-labile prodrugs of 5FU. Even if the drug was generated from all the precursors via palladium-mediated cleavage under biocompatible conditions, the propargyl group demonstrated superior sensitivity to palladium catalysis. The present study suggests that reaction kinetics is strongly influenced by the accessibility of the catalyst to the triple bond.

We have demonstrated that the pharmacological activity of 5FU can be “switched off” by alkylation chemistry of not only the *N*1 position but also the *N*3 position of 5FU. We have also shown that palladium-mediated *N*-propargylation occurs faster at position *N*1 than at position *N*3, and that pH can influence the reaction conversion rate. Bioorthogonal restoration of the drug's cytotoxic properties with either *N*1 or *N*3-propargylated 5FU by extracellular palladium chemistry in a range of cancer cells demonstrated that the rate of activation is an essential factor to rapidly achieve sustained and effective cytotoxic levels of drug. Overall, the studies reported herein indicate that the propargylation of 5FU's *N*1 position [masking strategy used by our group in the seminal work on palladium-activated prodrugs (Weiss et al., 2014a)] is yet the best approach available -in terms of bioorthogonality and palladium lability- to implement a BOOM-activated prodrug strategy with this particular chemotherapeutic drug.

### Conflict of interest statement

The authors declare that a patent is pending on the N-alkynyl derivatives of 5FU and its method of activation for medical use.
